# Partially ablative radiotherapy (PAR) for large mass tumors using simultaneous integrated boost: A dose‐escalation feasibility study

**DOI:** 10.1002/acm2.12427

**Published:** 2018-09-15

**Authors:** Savino Cilla, Francesco Deodato, Anna Ianiro, Gabriella Macchia, Vincenzo Picardi, Milly Buwenge, Silvia Cammelli, Alice Zamagni, Vincenzo Valentini, Alessio G. Morganti

**Affiliations:** ^1^ Medical Physics Unit Fondazione di Ricerca e Cura Giovanni Paolo II ‐ Università Cattolica del Sacro Cuore Campobasso Italy; ^2^ Radiation Oncology Unit Fondazione di Ricerca e Cura Giovanni Paolo II ‐ Università Cattolica del Sacro Cuore Campobasso Italy; ^3^ Radiation Oncology Department DIMES Università di Bologna ‐ Ospedale S.Orsola Malpighi Bologna Italy; ^4^ Radiation Oncology Department Policlinico Universitario A. Gemelli ‐ Università Cattolica del Sacro Cuore Roma Italy

**Keywords:** palliative, simultaneous integrated boost, VMAT

## Abstract

**Purpose:**

This study aimed to assess the feasibility to plan and deliver highly heterogeneous doses to symptomatic large tumors using volumetric modulated arc therapy (VMAT) and simultaneous integrated boost (SIB) during a short course palliative accelerated radiotherapy.

**Methods:**

A patient with a large symptomatic chordoma infiltrating the right gluteal region was selected. A modified SIB treatment was implemented to irradiate the central volume of the tumor (boost target volume, BTV) up to 10 Gy/fraction in a dose escalation trial while maintaining the remaining tumor volume (planning target volume, PTV) and the surrounding healthy tissues within 5 Gy/fraction in twice daily fractions for two consecutive days. Four SIB plans were generated in the dual‐arc modality; a basal dose of 20 Gy was prescribed to the PTV, while the BTV was boosted up to 40 Gy. For comparison purposes, plans obtained with a sequential boost (SEQ plans) were also generated. All plans were optimized to deliver at least 95% of the prescription dose to the targets. Dose contrast index (DCI), conformity index (CI), integral dose (ID), and the irradiated body volumes at 5, 10, and 20 Gy were evaluated.

**Results:**

At equal targets coverage, SIB plans provided major improvement in DCI, CI, and ID with respect to SEQ plans. When BTV dose escalated up to 200% of PTV prescription, DCI resulted in 66% for SIB plans and 37% for SEQ plans; the ID increase was only 11% for SIB plans (vs 27% for SEQ plans) and the increase in healthy tissues receiving more than 5, 10, and 20 Gy was less than 2%. Pretreatment dose verification reported a γ‐value passing rate greater than 95% with 3%(global)‐2 mm.

**Conclusion:**

A modified SIB technique is dosimetrically feasible for large tumors, where doses higher than the tolerance dose of healthy tissues are necessary to increase the therapeutic gain.

## 1 INTRODUCTION

Hypofractionated radiotherapy has been proved to be an effective treatment in local symptomatic control of advanced metastatic tumors, such as pain, bleeding, and obstruction.^1^ In the past years, some clinical evidence already suggested the use of shorter fractionation schedules having the same effectiveness as long‐course RT in symptom control of patients with incurable cancer.^1^, ^2^ For example, a fractionation regimen of twice‐daily fractionation (3.70 Gy/fraction twice daily) in 2 days, repeated two times at monthly intervals, was tested in the RTOG 8502 phase II trial for advanced pelvic cancer,^3^, ^4^ reporting a significant reduction of grade 3 and 4 late toxicity with respect to traditional treatments based on monthly repeated high single‐fraction doses (up to 10 Gy).

As palliative therapy should achieve symptomatic relief with the shortest possible timing, the efficacy of more rapid fractionation schemes was deeply investigated in the last years in our center. In particular, we assessed the tolerability of short‐course accelerated RT in twice daily fractions for two consecutive days in several dose‐escalation trials for head–neck, brain, and pelvic tumors.^5^, ^6^, ^7^, ^8^ Our results reported that treatments of twice daily fractions for two consecutive days (5 Gy/fraction) were well tolerated showing a high rate of symptom remission with a good impact on quality of life.

In the last decade, the advances in radiotherapy technology have greatly shown the potential to improve outcomes for patients. In particular, the introduction in clinical practice of intensity‐modulated radiotherapy (IMRT) and volumetric modulated arc therapy (VMAT) has greatly improved sparing of normal tissue and, hence, enabling dose escalation and/or intensified fractionation also in the case of large mass tumors.^9^, ^10^ Furthermore, IMRT and VMAT techniques allowed the simultaneous delivery of different doses to different target volumes within a single fraction, an approach called simultaneous integrated boost (SIB), that has proven to be more efficient in terms of treatment shortening and radiobiological improved effect.^11^, ^12^, ^13^ VMAT demonstrated remarkable capability to explore this flexibility thanks to its rotational delivery modality optimizing incident radiation from 360° around the patient to achieve highly conformal and heterogeneous doses.^14^ In addition, thanks to its rotational dose delivery and to the introduction of flattening filter‐free beams (enabling high‐dose rate irradiations), VMAT advantages included a large reduction of treatment delivery time, especially for radiosurgical doses.^15^

The application of these new advanced techniques to palliative treatments suggests a new possible clinical scenario. Traditionally, standard techniques were aimed to deliver a uniform dose to the tumor volume in order to reduce the tumor size and palliate the symptoms. In cases of large tumors the prescription of a homogeneous dose produces a significant irradiation of the surrounding organs and therefore limits the total dose administrable without serious side effects. But today, the use of advanced techniques as VMAT can allow the use of a dose boost to the central–internal region of the tumor, with the aim to increase tumor response and therefore the palliative effect, without significant increase of healthy tissue irradiation. In particular, if the dose boost is a stereotactic‐like dose level, that is, an ablative dose, a partial ablative radiotherapy (PAR) has the potential to amplify both the antineoplastic and palliative effects, not only by improving the cell killing almost in a tumor subvolume, but also by activating immunological antineoplastic mechanisms.^16^ This strategy constitutes a modified SIB technique whose main point, as opposed to the widely used SIB technique, is to deliver the highest possible dose to a boost volume, limiting the dose to the healthy tissue to the tolerance dose.^17^, ^18^

In this paper we planned a dose‐escalation study for a large tumor in the pelvic area to evaluate the feasibility of a short‐course accelerated RT based on this modified SIB technique, at five sequential dose levels.

## 2 MATERIALS AND METHODS

### 2.A Patient, volume definition and dose levels

**Figure 1:**
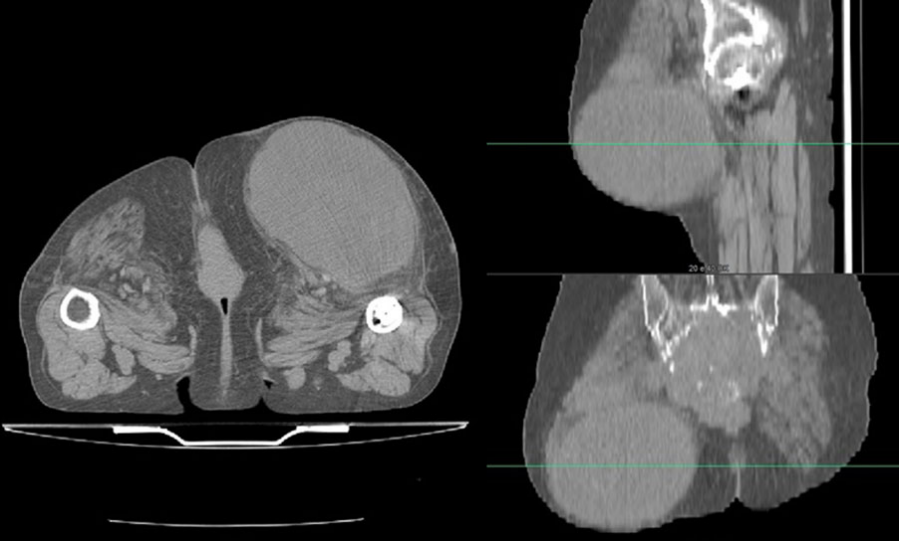
Axial, sagittal, and coronal CT scans of a huge chordoma, showing a large swelling infiltrating the right gluteal region and the ipsilateral thigh root.

For this dosimetric analysis we selected a patient with a large symptomatic tumor. A 65‐year‐old female patient with a huge chordoma was enrolled in this study and treated with modified SIB radiotherapy. The patient showed a large swelling infiltrating the right gluteal region and the ipsilateral thigh root (Fig. 1).

The lesion was considered unresectable for its local extension and the presence of lung metastases.

The patient underwent a computed tomography scan (Brilliance CT Big Bore, Philips, Netherlands) in prone position. The macroscopic extent of the tumor was defined as the gross tumor volume (GTV). A planning target volume (PTV) and a boost target volume (BTV) were defined as the gross tumor volume (GTV) plus 1 cm and minus 3.0 cm, respectively. For this patient, the PTV and BTV volumes were 1969.0 and 218.3 cm^3^, respectively. Two different doses were simultaneously delivered to the PTV and BTV according to a dose‐escalation protocol in four fractions. A basal dose of 20 Gy was prescribed to the PTV, while the BTV was boosted to 25, 30, 35, and 40 Gy. The aim was to irradiate the central part of the tumor up to 10 Gy/fraction while maintaining the border area of the tumor and the surrounding healthy tissues within 5 Gy/fraction.

### 2.B Treatment planning

All plans were created with the VMAT technique using the Oncentra MasterPlan treatment planning system v.4.1 (Elekta, Crawley, England) for 6‐MV beams from an Elekta Precise linear accelerator. The integrated MLC consists of 40 opposed pairs of leaves, with a projected width of 1 cm at the isocenter and no leaf interdigitation allowed. The beam data modeling in Oncentra MasterPlan was implemented with the following nominal values for VMAT specific parameters: a maximal gantry speed of 6°per s, with minimum and maximum MU per degree of gantry rotation equal to 0.1 MU per degree and 20.0 MU per degree, respectively. The fastest combination of dose rate, gantry speed, and leaf speed was automatically selected by the linac control system software Precise Desktop 7 during the arc delivery.

Four plans were optimized with the SIB strategy (SIB20/25, SIB20/30, SIB20/35, and SIB20/40) and generated with the “dual‐arc” feature, using the optimization process described previously.^9^ The planner defines the gantry rotation direction, the start–stop angles and the gantry spacing to define the control points (CP). Then the optimization begins generating coarse initial CP with fluence maps at the start and stop angles and at 24° increments from the start angle, and subsequent MLC sequencing generating two segments per gantry angle. The segments are subsequently then spread out evenly and cloned to achieve the required gantry angle spacing. All CP are processed to fulfill the motion constraints: maximum leaf speed, dose rates, and delivery times. A direct machine parameter optimization is performed on all CP considering all machine and user constraints, followed by a final dose calculation and segment weight optimization. For all plans, an entire gantry rotation was described in the optimization process by a sequence of 90 control points, that is, every 4°. Collimator was set at 10° to minimize the tongue‐and‐groove cumulative effect. Dose calculation was performed using the collapsed cone convolution algorithm and a dose‐grid resolution of 2 × 2 mm^2^ in the axial plane.

For comparison purposes, plans obtained with a sequential boost (SEQ20/25, SEQ20/30, SEQ20/35, and SEQ20/40) were generated using one arc for each target. All plans were optimized to deliver at least 95% of the prescription dose to the PTV. For the BTV, the goal was to deliver the boost dose to at least 95% of the volume, penalizing volumes receiving more than 107% of prescription dose.

In particular, a priority goal was to enhance the steepness of dose gradient outside the target volume. In MasterPlan optimization module, this task can be effectively performed using the so‐called “surrounding dose fall‐off” objective.^19^ This objective is able to control the rate of dose fall‐off within a structure (e.g., the patient body), penalizing doses above a certain level at a certain distance to the target. In other words, this function is a linearly decreasing dose level starting at the high dose level adjacent to the target and dropping to the low‐dose level at a defined distance from the target. In this study, the surrounding dose fall‐off objective was used in order to potentially decrease the dose from 20 to 10 Gy in a 4‐cm distance.

### 2.C Plans analysis and evaluation

#### 2.C.1 Dose contrast and biological dose contrast indexes

To quantify the ability to deliver highly heterogeneous doses as requested for SIB (minimizing the high doses to elective regions), a metric called dose contrast (DC) index was used.^20^ The ideal DC (iDC) was defined as the ratio between the prescription doses to the BTV and the PTV. As the delivery of higher doses to the BTV increases doses to the surrounding PTV, we defined an actual DC (aDC) as the mean dose to the BTV divided by the mean dose to the PTV (excluding BTV). The ratio of iDC and aDC defines the normalized dose contrast (nDC) and quantifies the deviation of the actual aDC from the ideal iDC. A nDC value closer to 1 indicates a better dose contrast.

Similarly, we introduced a biological dose contrast (BDC) index as the biological equivalent dose (BED) of the average BTV dose divided by that of the PTV dose. BED was calculated by

$$\text{BED}=nd\cdot \left(1+\frac{d}{\alpha /\beta }\right)$$

where *d* is the fractional dose and *n* is the number of fractions. An α/β value of 10 was assumed for the tumor tissue. The BED to PTV was equal to 30 Gy; the BED to the PTV25, PTV30, PTV35, and PTV40 were equal to 40.6, 52.5, 65.6, and 80 Gy, respectively. The BED contrast was defined similarly as the ratio of BED to the BTV and to the PTV. The normalized BED contrast (nBDC) was defined similarly as the ratio of actual BED contrast and ideal BED contrast.

#### 2.C.2 Conformity index

Conformity indexes (CIs) were defined as the volume encompassed by the 95% isodose divided by the PTV volumes.^21^ A CI value closer to 1 indicates a more conformal dose distribution to PTV.

#### 2.C.3 Integral dose and healthy tissue irradiation

Integral dose (ID) is the volume integral of the dose deposited in the patient body and is equal to the mean dose times the volume irradiated to any dose (excluding the PTV).^22^ The ID was then used to evaluate the cost to deliver highly heterogeneous dose to the BTV. This was reported together with the irradiated volumes at the dose levels of 5, 10, and 20 Gy (V5, V10, and V20).

### 2.D Dosimetric verification

Last, a detailed dosimetric verification of the plans was performed in order to assess the technical feasibility of this treatment. The absolute doses were measured utilizing the 2D‐array seven29 ion‐chamber array and the Octavius phantom^23^ (PTW, Freiburg, Germany). The ion‐chamber array consists of a matrix of 729 cubic vented ionization chambers with 0.5 × 0.5 cm cross section, spaced 1 cm center‐to‐center, giving a total area of 27 × 27 cm^2^. The Octavius phantom, designed to allow VMAT plan verification thanks to a cavity that houses the 2D‐array, has an octagonal shape and it is made of polystyrene (physical density of 1.04 g/cm^3^). Plans were recalculated on phantoms representing the Octavius geometry and density; the doses were measured on both coronal and sagittal planes for every arc. This methodology represents a true composite QA method since all the radiation beams are delivered to a stationary measurement device in a phantom placed on the couch using the actual patient treatment beam geometry (MUs, MLC leaf positions, jaws, gantry).

Comparison of measured versus calculated dose distributions was done with the Verisoft software v4.0 (PTW, Freiburg, Germany) by means of the gamma evaluation. This last was based on the theoretical concept introduced by Low et al.,^24^ using the approach of Depuydt et al.^25^ to take into account practical considerations concerning the discrete nature of data. The pass rate of γ‐analysis (γ%) was computed by comparing the calculated and measured dose distributions using 3%/3 mm, 3%/2 mm, and 2%/2 mm criteria, respectively. Both global and local normalizations were used. A dose threshold was set to 10% to exclude low‐dose areas that have no or little clinical relevance but can significantly bias the analysis.

## 3 RESULTS

**Figure 2:**
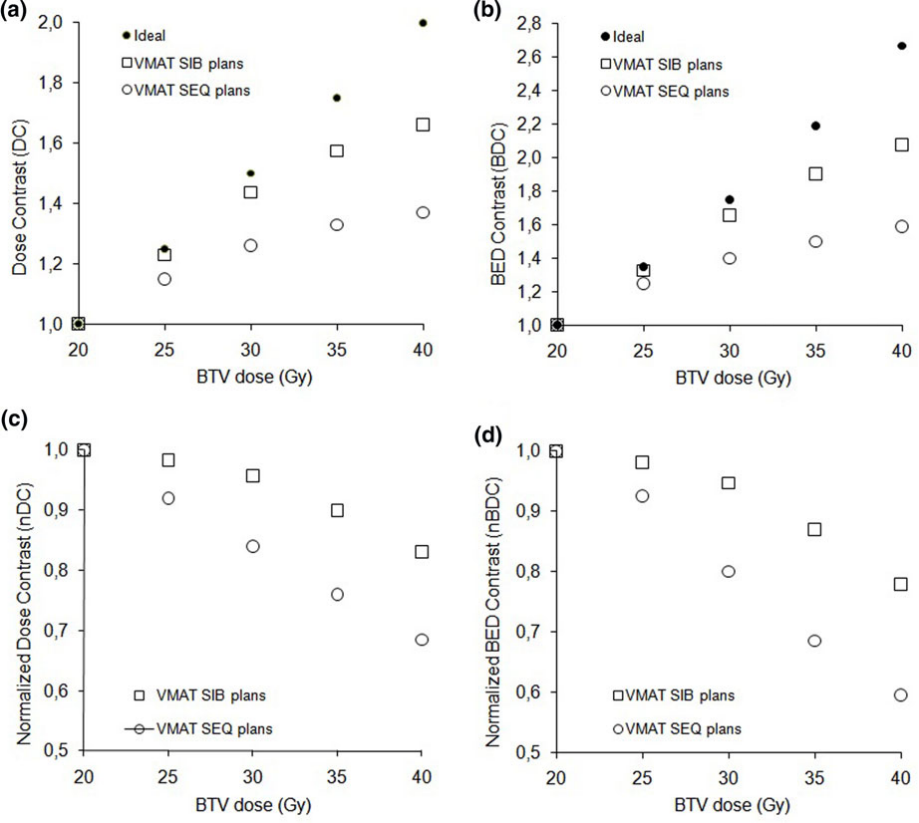
Dose contrast (a), BED contrast (b), normalized dose contrast (c), and normalized BED contrast (d) indexes of SIB and SEQ plans as a function of BTV dose.

All plans satisfied the target dose coverage objectives. Figure 2 shows the dose contrast indexes of SIB and SEQ plans as a function of BTV dose. Both DC and BDC increased with increasing boost dose [Figs. 2(a) and 2(b)]. Also the deviations from the ideal contrast indexes (nDC and nBDC) increased as a function of BTV dose [Figs. 2(c) and 2(d)]. SIB plans reported a major improvement in dose contrast with respect to SEQ plans. In particular, the DC and BDC increase resulted in 66% and 108% when the BTV prescription reached 200% of the basal dose (40 Gy), compared to the 37% and 59% increase for the SEQ plans, respectively.

**Figure 3:**
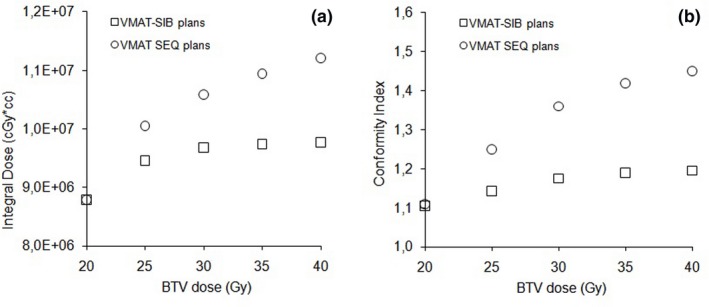
Integral dose to normal tissue (a) and conformity index (b) as a function of boost dose for the SIB and SEQ plans.

Figure 3(a) shows the integral dose to normal tissue as a function of boost dose. For the SIB plans, the percentage increase in integral dose to the healthy tissues was 11.1% when the BTV dose was escalated up to 200% of basal dose. In particular, the integral dose remains approximately constant, for example, within 3%, with increasing boost doses from 25 to 40 Gy. For comparison purpose, SEQ plans resulted in systematically higher integral dose that linearly increased with higher boost dose, up to 27.4% of the basal plan. Figure 3(b) shows the conformity indexes as a function of boost dose. As well as for ID, for SIB plans the conformity indexes show only a slight increase with boost dose: when BTV dose escalated up to 200% of PTV prescription, the CI increase was less than 9% (to be compared with a 31% increase for SEQ plan). The increase in healthy tissues receiving more than 5, 10, and 20 Gy was less than 2%.

**Figure 4:**
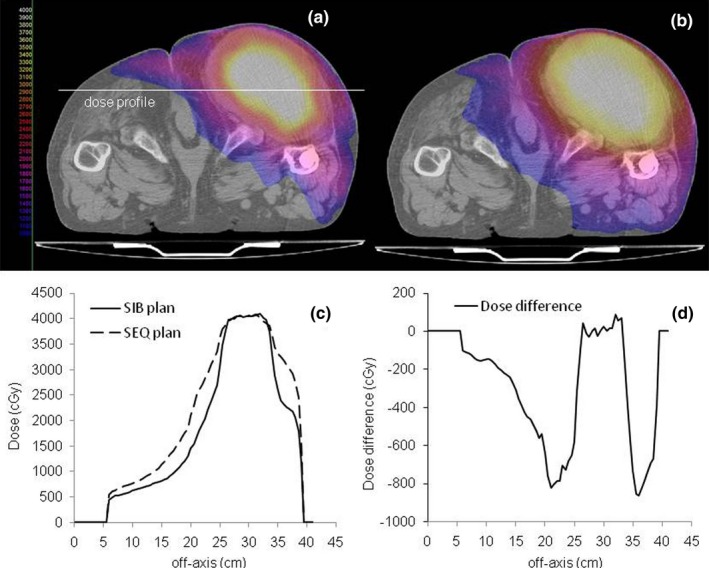
Differences in dose distributions between SIB (a) and SEQ (b) plans visualized in axial images for the higher dose level. (c) reports the comparison of two dose profiles [along the line drawn in (a)] for SIB and SEQ plans, respectively. (d) shows the dose difference along the profile.

In Figs. 4(a) and 4(b), the differences between SIB and SEQ plans are visualized in axial isodose images for the higher dose level. The SEQ plan shows an evident higher dose spillage from the BTV to the PTV and from the PTV to healthy tissues.

Figure 4(c) reports the comparison of two dose profiles (along the line drawn in Fig. 4(a) for SIB and SEQ plans, respectively. Figure 4(d) shows the dose difference along the profile.

**Figure 5:**
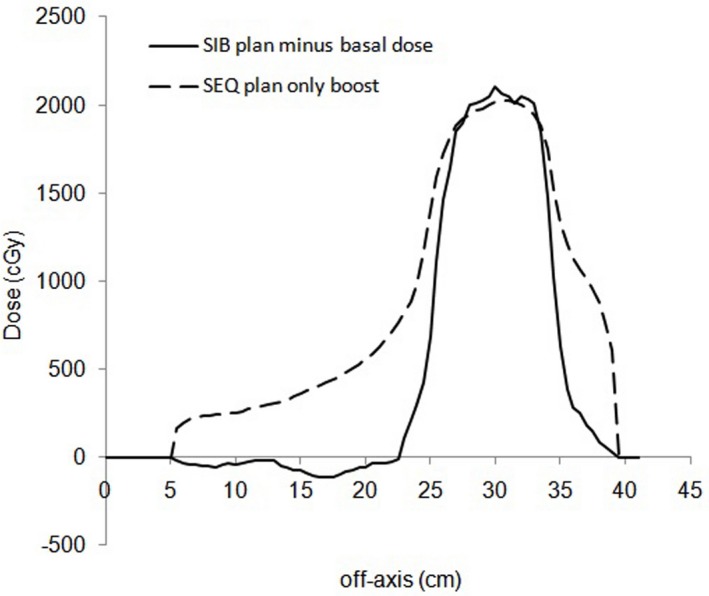
Black solid line represents the dose profile from the SIB plan with basal dose subtracted. The dashed line represents the dose profile from the PTV‐only SEQ plan.

Figure 5 shows the comparison between the dose profile of SIB plan when the 20 Gy basal dose is subtracted from the plan (solid line) and the dose profile of SEQ plan obtained for the boost BTV only (dotted line). The figure clearly shows the major improvement of dose gradient and the steeper penumbra around the BTV for the SIB plan.

To better quantify the dose distribution differences between SEQ and SIB plans, Table 1 reports the absolute volumes of the isodose clouds receiving 5 Gy, 10 Gy, 15 Gy, and 19 Gy (the 95% of prescribed dose at basal level).

**Table 1:** Absolute volumes of the isodose clouds receiving 5, 10, 15, and 19 Gy (the 95% of prescribed dose at basal level) for SEQ and SIB plans, respectively

	V_5Gy_ (cc)	V_10Gy_ (cc)	V_l5Gy_ (cc)	V_l9Gy_ (CC)
Basal plan	8158	4554	2992	2149
SIB plans				
SIB20/25	8261	4822	3203	2245
SIB20/30	8356	4967	3301	2311
SIB20/35	8474	5063	3333	2331
SIB20/40	8572	5103	3350	2354
SEQ plans				
SEQ20/25	8633	5020	3312	2454
SEQ20/30	9159	5291	3498	2661
SEQ20/35	9645	5506	3661	2767
SEQ20/40	10,021	5775	3783	2844

An accurate pretreatment verification was performed for three plans of increasing complexity (the basal plan, SIB20/30, and SIB20/40). Each arc was delivered two times, once in coronal and then in sagittal plane, so that each plan has four measurements. Table 2 shows the mean and range of the gamma passing rate (γ%) for each plan obtained using different acceptance criteria (2%/2 mm, 3%/2 mm 3%/3 mm) with both local and global normalization. When the commonly used 3%/3 mm criterion was considered, γ% was above 98% and 90% for all plans using global and local normalization, respectively. The γ% was observed to decrease as the criteria became more stringent. In addition, there is a clear tendency that the gamma passing rate decreases with increasing plan complexity, for example, as the boost dose increases. However, also with the stringent 2%(global)/2 mm criterion, the mean passing rate achieved an acceptable value of at least 94% for all plans.

**Table 2:** Mean and range of the gamma passing rate for each plan obtained using different acceptance criteria (2%/2 mm, 3%/2 mm 3%/3 mm) with both local and global normalization

	MU	γ passing rate (%)					
		2%/2 mm		3%/2 mm		3%/3 mm	
		Global	Local	Global	Local	Global	Local
Basal	1411	96.8 (96.5–97.6)	87.1 (85.1–91.1)	98.2 (97.7–98.6)	91.6 (89.2–96.4)	100.0 (99.8–100.0)	94.6 (93.2–98.3)
SIB20/30	1691	95.9 (94.6–97.6)	85.5 (83.1–90.7)	97.7 (97.4–98.4)	87.0 (79.1–94.2)	99.5 (99.3–100.0)	92.3 (90.8–94.0)
SIB20/40	2027	94.8 (94.0–97.1)	81.0 (76.3–84.3)	96.5 (95.2–98.5)	82.8 (76.5–86.2)	98.2 (98.5–98.6)	91.4 (90.2–92.3)

**Figure 6:**
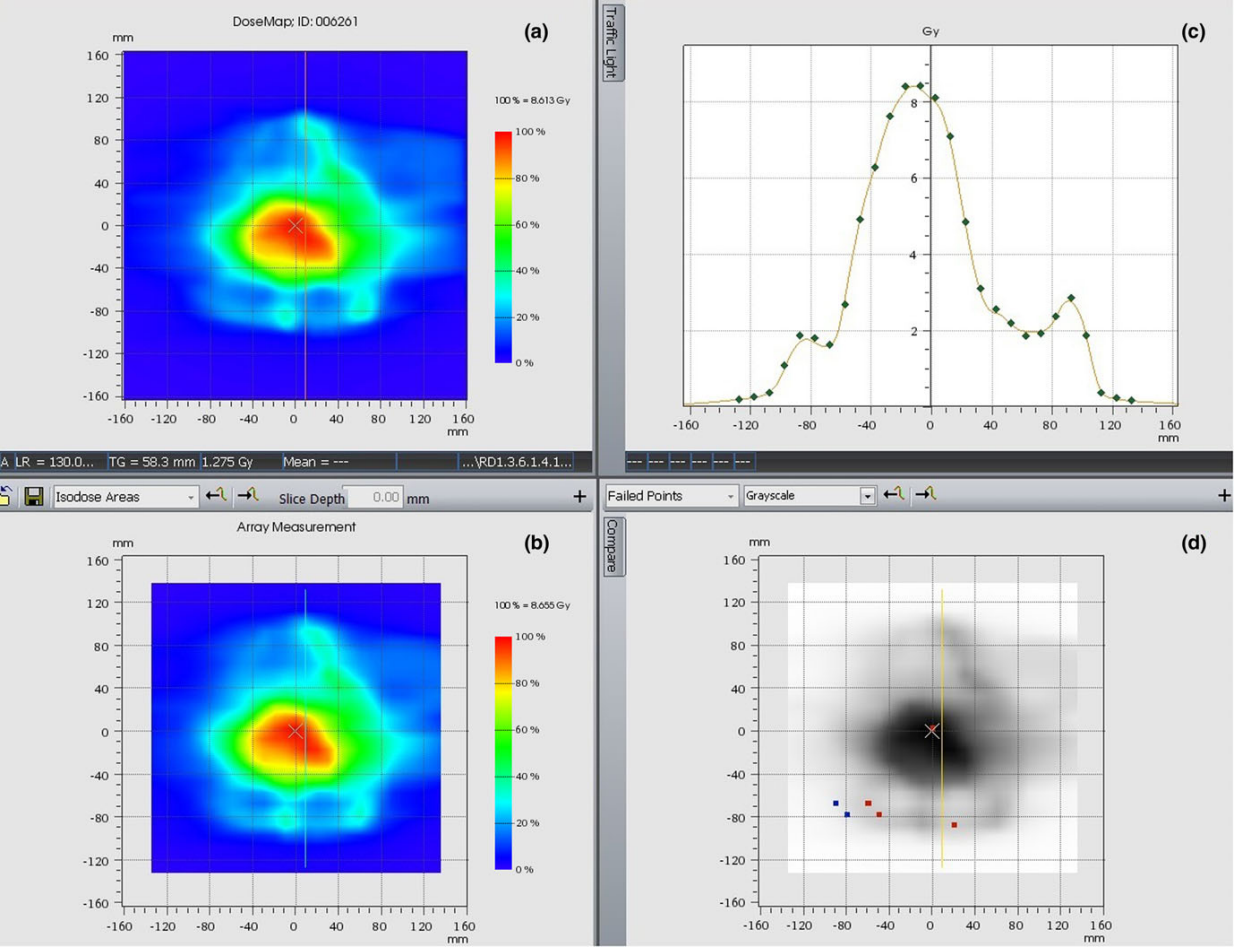
Gamma evaluation results for the SIB20/40 plan in the sagittal plane, reporting the calculated dose distribution (a), the measured dose distribution (b), the calculated versus measured cross‐plane profile, (c) and the failed points distribution (d).

Figure 6 shows an example of gamma evaluation results for the SIB20/40 plan in the sagittal plane, reporting the calculated dose distribution (a), the measured dose distribution (b), the calculated versus measured cross‐plane profile, (c) and the failed points distribution (d).

## 4 DISCUSSION

The management of large bulky tumors as chordomas or sarcomas is challenging. These tumors often present a large burden at the time of diagnosis, growing along critical bony and neural structures, and preventing surgical resection in about half of the cases.^26^ In addition, these tumors are relatively resistant to conventional photon‐based radiotherapy, with high rates of local recurrences.^27^ Then, high biological equivalent doses are needed to achieve greater local control rates, with some studies suggesting that biological equivalent doses greater than >70 GyRBE (proton dose unit, gray relative biological effectiveness) are efficacious both as an adjuvant to surgery and as definitive therapy.^27^, ^28^ However, the delivery of high doses to large tumors is problematic due to the major increase in healthy tissue integral dose which can result in serious side effects. In the last year, new treatment modalities have been proved to deliver high radiobiologically effective treatments in patients with large tumors. Recently, intensity modulated proton therapy (IMPT) and carbon ions therapy reported clear evidences for high local control rates in chordomas, providing an effective alternative to traditional photon radiotherapy.^28^, ^29^ For example, a recent study focusing on the outcomes and responses of primary sacral chordomas in 33 patients treated with protons at 70.4 GyRBE doses in 32 fractions reported a 3‐year local progression‐free survival, distant metastasis‐free survival, disease‐free survival, with an overall survival rate of 89.6%, 88.2%, 81.9%, and 92.7%, respectively.^30^

Modern radiation therapy techniques have also allowed non conventional approaches to obtain highly heterogenous doses within a large tumor volume. This technique, called “Lattice”, represents an evolution of the older high‐dose GRID radiotherapy^31^ and allows for localized 3D high‐dose array within the tumor volume, in which highly concentrated hot‐spot doses are located in lattice vertices with a rapid dose fall‐off between them, then resulting in a periodic three‐dimensional peak‐to‐valley dose distribution.^32^ Recent research in radiobiology has provided a theoretical basis using the concept of bystander effect within the parts of irradiated tumor volume not directly irradiated.^33^ In addition, a robust abscopal effect in distant tumors or metastatic lesions that are not directly treated have been also described.^34^ All these data strongly suggest that the use of very high heterogeneous doses could induce a higher rate of tumor cell apoptosis in bulky and hypoxic tumors than conventional radiotherapy.

Unfortunately, these selected complex techniques are not readily available and are present in very few Centers. On the contrary, in the last decade VMAT had a widespread worldwide implementation for most of anatomical sites due to highly conformal dose distributions with improved target volume coverage and sparing of normal tissues compared with conventional radiotherapy, thus representing an optimal platform for SIB planning and delivering.^35^, ^36^ SIB strategy have been recently also applied to the treatment of several types of bulky tumors, such as soft tissue sarcoma,^37^ esophageal cancer^38^, and lung cancer,^39^ with encouraging results in terms of tumor volume reduction and prolonged progression‐free survival.

In the present study, we implemented a modified SIB strategy, focused on intratumor dose escalation, with the aim to deliver an ablative fraction dose size to the central region of a bulky tumor (up to 10 Gy/fraction) together with a palliative dose fraction size (5 Gy/fraction) to the peripheral zone of the irradiating tumor. This way, for a twice daily fraction for two consecutive days, the equivalent dose in 2 Gy/fraction (EQD2 Gy) to the central part of the tumor was calculated to be 66.7 Gy, while the EQD2 Gy of the surrounding healthy tissue was calculated to be 25.0 Gy. The aim was to obtain a major increase of the therapeutic gain due to double benefits for bulky tumors: an effective high tumor control with negligible treatment toxicities.

Using the dose contrast index concept, results showed that the SIB plans allowed the delivery of additional dose to the BTV with a minimal increase of dose to surrounding tissues, with respect to SEQ plans. This behavior can be clearly show in Fig. 5 that reports the comparison between the dose profile of the SIB plan when the boost prescription reached 200% of PTV prescription and the 20 Gy basal dose is subtracted and the dose profile with the BTV only boost dose from SEQ plan. SIB plan not only has a much steeper dose fall‐out around the BTV but it also exhibits a negative dose around the BTV. The theoretical demonstration that integrating boost dose into the original plan and optimizing in a single solution domain has the potential to deliver a negative dose in the region around the BTV (by subtracting from the basal dose to the PTV) has been well explained by Ahnesjo et al.^40^

Another relevant result obtained in this study is that SIB plans provided an integral dose relatively constant as a function of boost dose. In particular, we demonstrated that integral dose remains approximately constant with increasing boost doses from 25 to 40 Gy. This result enforces the choice of a significant boost dose to increase local control and alleviate symptoms because, in this context, the integral dose to healthy tissues do not represents a limiting factor. Recently, a similar approach has been proposed for the management of a breast cancer and large abdominal tumors.^17^, ^18^

An excellent agreement (more than 95%) between the measured and calculated dose distributions for the 3%(global)/2 mm criterion was found for all plans, regardless of plan complexity. These results comply the suggestions of the recent AAPM task group no. 218 report,^41^ focused on tolerance limits and methodologies for IMRT measurement‐based verification QA. This report recommended as universal tolerance limits a γ‐passing rate ≥95%, with 3%/2 mm criterion, a 10% dose threshold and using global normalization in absolute dose. However, the utility of common adopted γ‐passing rate for the purpose of patient‐specific dose QA has been recently questioned, since this method may lack sensitivity and specificity in predicting clinically important patient dose errors.^42^ We then performed a more stringent γ‐analysis, restricting traditional tolerances; despite stricter discrimination in terms of dose difference/distance to agreement, our results confirm the deliverability of our modified SIB‐VMAT technique and its reliability and safety for clinical applications.

## 5 CONCLUSIONS

We introduced a modified SIB technique having the potential to be particularly effective for large bulky tumors, where doses higher than the tolerance dose of healthy tissues are necessary to increase the therapeutic gain. We showed that despite the major dose escalation in the boost volume, the dose conformity to PTV and the integral dose to the normal tissue minimally increased, with a dose spillage from PTV to normal tissue almost constant. The pretreatment dose verification supplied an excellent agreement with calculated values ensuring the accuracy of delivered dose distribution in clinical cases. The safe delivery of ablative dose in the central part of the tumor is feasible and has the potential to greatly improve the palliative effect.

## CONFLICT OF INTEREST

The authors declare no conflict of interest.
